# Identification of a novel antimicrobial peptide from amphioxus *Branchiostoma japonicum* by *in silico* and functional analyses

**DOI:** 10.1038/srep18355

**Published:** 2015-12-18

**Authors:** Haohan Liu, Miaomiao Lei, Xiaoyuan Du, Pengfei Cui, Shicui Zhang

**Affiliations:** 1 Laboratory for Evolution & Development, Institute of Evolution & Marine Biodiversity and Department of Marine Biology, Ocean University of China, Qingdao 266003, China

## Abstract

The emergence of multi-drug resistant (MDR) microbes leads to urgent demands for novel antibiotics exploration. We demonstrated a cDNA from amphioxus *Branchiostoma japonicum*, designated *Bjamp1*, encoded a protein with features typical of antimicrobial peptides (AMPs), which is not homologous to any AMPs currently discovered. It was found that *Bjamp1* was expressed in distinct tissues, and its expression was remarkably up-regulated following challenge with LPS and LTA. Moreover, the synthesized putative mature AMP, mBjAMP1, underwent a coil-to-helix transition in the presence of TFE or SDS, agreeing well with the expectation that BjAMP1 was a potential AMP. Functional assays showed that mBjAMP1 inhibited the growth of all the bacteria tested, and induced membrane/cytoplasmic damage. ELISA indicated that mBjAMP1 was a pattern recognition molecule capable of identifying LPS and LTA. Importantly, mBjAMP1 disrupted the bacterial membranes by a membranolytic mechanism. Additionally, mBjAMP1 was non-cytotoxic to mammalian cells. Collectively, these data indicate that mBjAMP1 is a new AMP with a high bacterial membrane selectivity, rendering it a promising template for the design of novel peptide antibiotics against MDR microbes. It also shows for the first time that use of signal conserved sequence of AMPs is effective identifying potential AMPs across different animal classes.

The emergence of multi-drug resistant (MDR) microbes caused by overuse of antibiotics has resulted in the less efficacy of major antimicrobial drugs used in clinical settings^1^, which has become an increasingly serious problem globally, leading to urgent demands for exploration of novel antibiotics, such as phytochemicals, synthetic antibiotics, antimicrobial peptides (AMPs), and inhibitors for drug-efflux pumps^2^,^3^,^4^,^5^,^6^,^7^,^8^. AMPs are endogenous antibiotics that are widely distributed in nature as ancient components of innate immunity. They are often cationic and amphipathic molecules that interact with microbial membranes, and kill microbes by direct disruption of cellular components, including the microbial membrane and DNA^9^,^10^, and thus the acquisition of resistance against AMPs is very rare, compared to conventional antibiotics^11^. Accordingly, AMPs have attracted great attention for overcoming MDR microbes.

Currently, over 2,300 AMPs have been isolated and characterized according to the online updated Antimicrobial Peptide Database (APD)^12^,^13^. AMPs are usually small, gene-coded polypeptides that can be constitutively expressed or induced to fend off invading microbes. They can be isolated from natural sources, such as the skin mucosa of aquatic animals, a rich source of AMPs, but their isolation and characterization can be time-consuming and laborious^14^. In addition, it entails obtaining often exotic animals or their tissues in sufficient quantities, and pursuing peptides that may be produced only in small quantities, need to be induced or are present as inactive precursors, complicating assay-based identification methods. An alternative approach is to identify the genes encoding AMPs, either by directly isolating genomic DNA from small tissue samples, or by mining the vast amount of sequence information already deposited in genomic or expressed sequence tag (EST) databases^15^.

Identifying novel AMPs in databases largely depends upon the existence of a sufficient sequence homology and a query sequence from a known AMP. However, homology between orthologous AMPs is extremely lower because they are at the interface between the host and a complex and ever changing microbial biota, and are thus under strong positive selection for variation in many animal taxa^16^, resulting in significant divergence between orthologous AMPs of even closely related species. Fortunately, AMPs generally include signal sequences and proregions that tend to be significantly more conserved than mature AMPs or full-length AMPs themselves. This advantage, i.e. signal sequence conservation, has been successfully employed by Tessera *et al.*^15^ and Juretić *et al.*^17^ to search for and identify novel AMPs from databases within the same lineages of fish and amphibians, individually. Whether such an approach is applicable to animals from different lineages of more divergent species has not yet been explored to date. Thus, this study was carried out to test the possibility via using the signal sequence of jawless hagfish HFIAP-1, a known AMP of the cathelicidin family, as a query to search for AMP candidates from amphioxus databases. Here we report the identification of an uncharacterized AMP from amphioxus *Branchiostoma japonicum*, named as BjAMP1, its bactericidal activity and modes of action. This provides the first example that use of signal conserved sequence of AMPS is effective identifying potential AMPs across different animal classes.

## Results

### *Bjamp1* codes for a novel putative AMP

**Figure 1:**
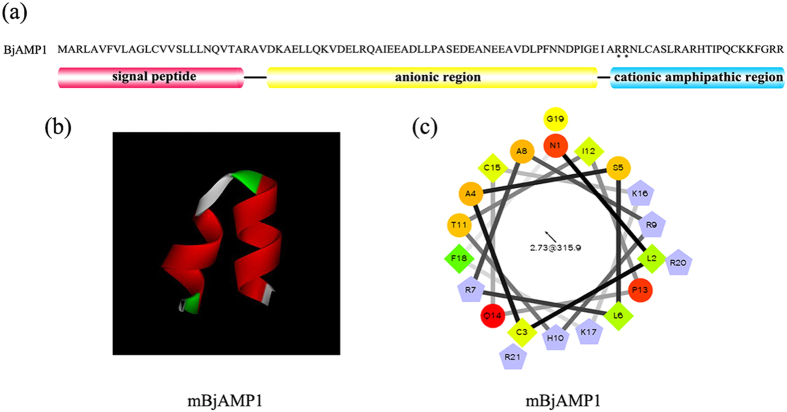
Amino acid composition distribution, 3D molecular modeling and helical wheel projection. (**a**) Amino acid composition distribution of BjAMP1. N-terminus is a signal peptide of 24 amino acid residues, followed by an anionic-rich region and a cationic-rich C-terminus extension. The putative dibasic cleavage site (^75^RR^76^) is indicated by (*). (**b**) 3D structure of mBjAMP1. (**c**) The helical wheel projection of mBjAMP1, demonstrating amphipathic potential of the peptide structure. Residues are numbered starting from the N-terminus. Hydrophilic residues are presented as circles, hydrophobic residues as diamonds, potentially negatively charged as triangles, and potentially positively charged as pentagons. Hydrophobicity is color coded: the most hydrophobic residue is green, and the amount of green is decreasing proportionally to the hydrophobicity, with zero hydrophobicity coded as yellow. Hydrophilic residues are coded red with pure red being the most hydrophilic (uncharged) residue, and the amount of red decreasing proportionally to the hydrophilicity. The potentially charged residues are light blue.

The application of a signal peptide from a jawless species (HFIAP-1 from Atlantic hagfish) as a query in a BLASTP search of protein databases for *B. floridae* resulted in 3 hits. When these hits were used as queries in a TBLASTN search of EST databases for *B. floridae*, it resulted in identification of only BW801384.1, which on translation showed characteristics of an AMP including cationic and amphipathic amino acid distribution (Fig. 1a). Notably, the homologue of BW801384.1, *Bjamp1* (Accession number in Genbank: KR779875), was isolated from another species of Cephalochordata, *B. japonicum*. The open reading frame (ORF) of *Bjamp1* was 294 bp long, encoding a protein of 97 amino acids (Suppl. Fig. 1) with a molecular mass of about 10.8 kDa and an isoelectric point (pI) of about 5.1. Moreover, the predicted proteins both comprised an N-terminal signal peptide of 24 amino acids, followed by an anionic region, and a C-terminal cationic extension (Fig. 1a), resembling typical precursors of known AMPs including HFIAP-1, a member of cathelicidin family. Contrasting to the cathelicidin family AMPs that include a cathelin-like domain containing four highly conserved cysteine residues^18^,^19^,^20^, no cathelin-like domain was identified in the putative AMPs, BjAMP1. This suggested that BjAMP1 may represent a novel putative AMP in Cephalochordata, which is different from the cathelicidin family, although they share a similar signal sequence.

Sequence alignment indicated that BjAMP1 was 94.8% identical to translated BW801384.1, with only 5 out of 97 residues being different (Suppl. Fig. 2). Analyses by PeptideCutter and CAMP revealed the presence of a dibasic cleavage site ^75^RR^76^, common in AMPs^20^,^21^,^22^, in the C-terminal region of BjAMP1 (Fig. 1a), which implicates the release of mature AMP. The predicted mature AMP, mBjAMP1, consisted of 21 residues, with a molecular mass of about 2.5 kDa and a net charge of +6. The 3D modeling showed that mBjAMP1 comprised 2 α-helices (Fig. 1b). Analysis by helical wheel projection indicated that mBjAMP1 had the properties to adopt an amphipathic α-helix structure (Fig. 1c), with a total hydrophobic ratio of 38%. These physicochemical and structural properties of mBjAMP1 well fit into the characteristics of AMPs. Moreover, comparison of mBjAMP1 with all the sequences in the online updated Antimicrobial Peptide Database APD (http://aps.unmc.edu/AP/main.php) revealed that they showed less than 38.5% similarity (<25% identity) to known AMPs (Suppl. Table 1). Therefore, mBjAMP1 is likely a novel AMP, which was thus identified functionally in the following experiments.

### Challenge with LPS and LTA stimulates expression of *Bjamp1*

**Figure 2:**
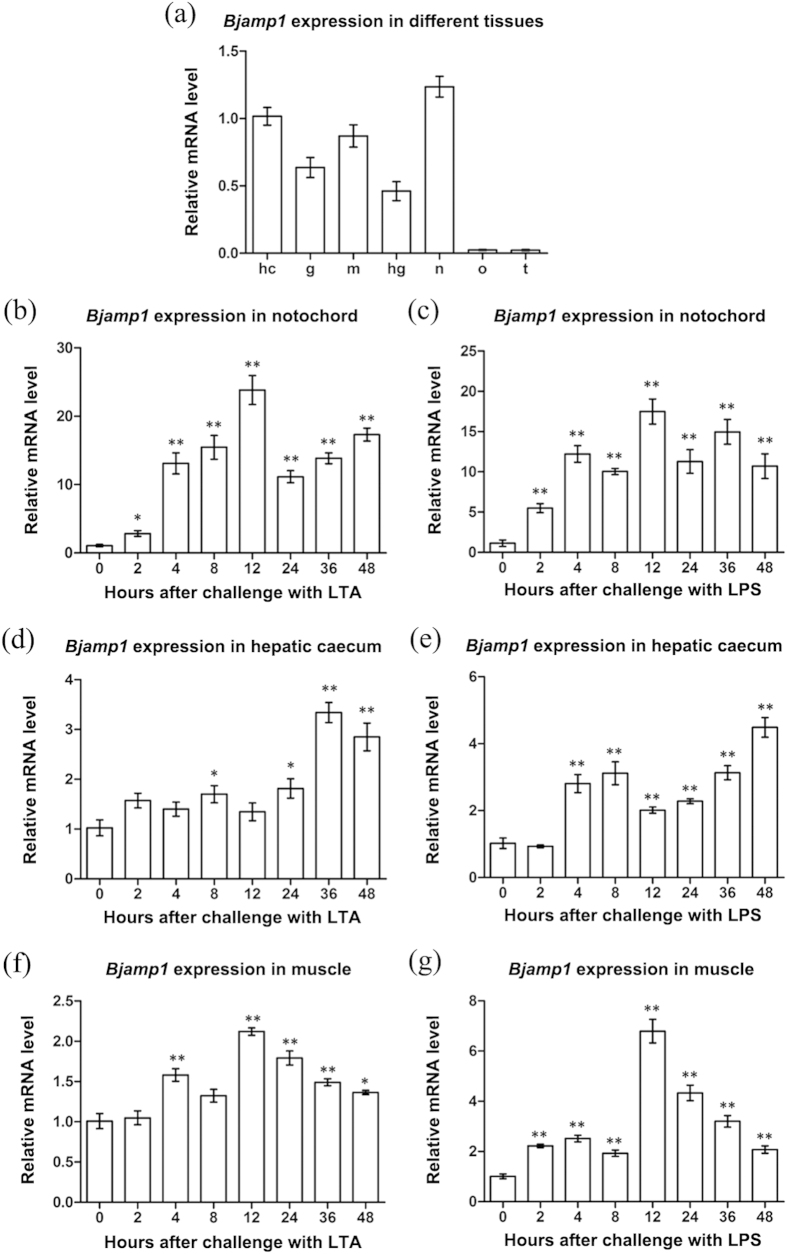
Tissue-specific as well as LPS- and LTA-induced expression of *Bjamp1*. (**a**) Total RNA was extracted from various tissues of *B. japonicum*, and the expression profiles of *Bjamp1* were determined in the different tissues by qRT-PCR. The expression level in the hepatic caecum was set as 1. Data were shown as mean ± SEM. hc, hepatic caecum; g, gill; m, muscle; hg, hind-gut; n, notochord; o, ovary; t, testis. (**b–g**) *B. japonicum* were sampled at 0, 2, 4, 8, 12, 24, 36 and 48 h after challenge with LPS or LTA, and total RNAs were extracted from notochord, hepatic caecum and muscle. The expression profiles of Bjamp1 was determined by qRT-PCR. In each case, the expression level of 0 hour was set as 1. Values were shown as mean ± SEM (n = 3). The symbol * indicates a significant difference (*p* < 0.05), and the symbol ** a extremely significant difference (*p* < 0.01).

Quantitative real-time PCR (qRT-PCR) was used to examine the expression of *Bjamp1* in *B. japonicum*. The dissociation curve of amplification product showed a single peak, indicative of specific amplification (Data not shown). Normally, *Bjamp1* was predominantly expressed in the gill, hepatic caecum, hind-gut, muscle and notochord, while little expression was observed in the ovary and testis (Fig. 2a), indicating that *Bjamp1* is expressed in a tissue-specific fashion. Challenge with lipopolysaccharide (LPS) and lipoteichoic acid (LTA) resulted in a significantly enhanced expression of *Bjamp1* in all the tissues tested, including the hepatic caecum, notochord and muscle (Fig. 2b–g). These suggested that BjAMP1 may play a role in the immune responses in *B. japonicum*, agreeing with our expectation that BjAMP1 was a molecule with features of AMPs.

### Circular dichroism (CD) spectroscopy

**Figure 3:**
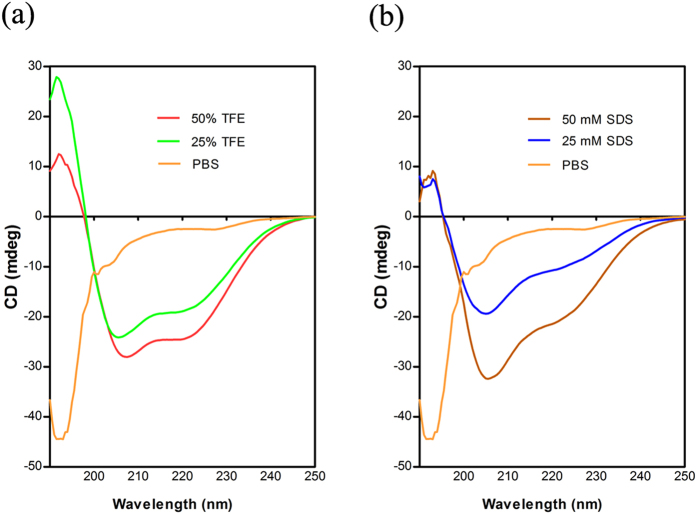
Circular dichroism spectra of mBjAMP1 (100 μg/ml) in the presence of TFE (**a**) and SDS (**b**). mBjAMP1 showed spectra consistent with random coil structure in PBS, and addition of increasing amounts of TFE and SDS resulted in an increasing conformational transition to an α-helical conformation.

The sequence of mBjAMP1 synthesized using standard solid-phase FMOC method was confirmed by MS/MS (Suppl. Fig. 3) The structural characteristics of mBjAMP1 in phosphate-buffered saline (PBS) with or without 2,2,2-trifluoroethanol (TFE) and sodium dodecyl sulfate (SDS) were determined using CD spectroscopy. As shown in Fig. 3, mBjAMP1 showed spectra consistent with random coil structure in PBS. Addition of increasing amounts of TFE and SDS clearly resulted in an increasing conformational transition to an α-helical conformation.

### mBjAMP1 is a bactericidal agent

**Figure 4:**
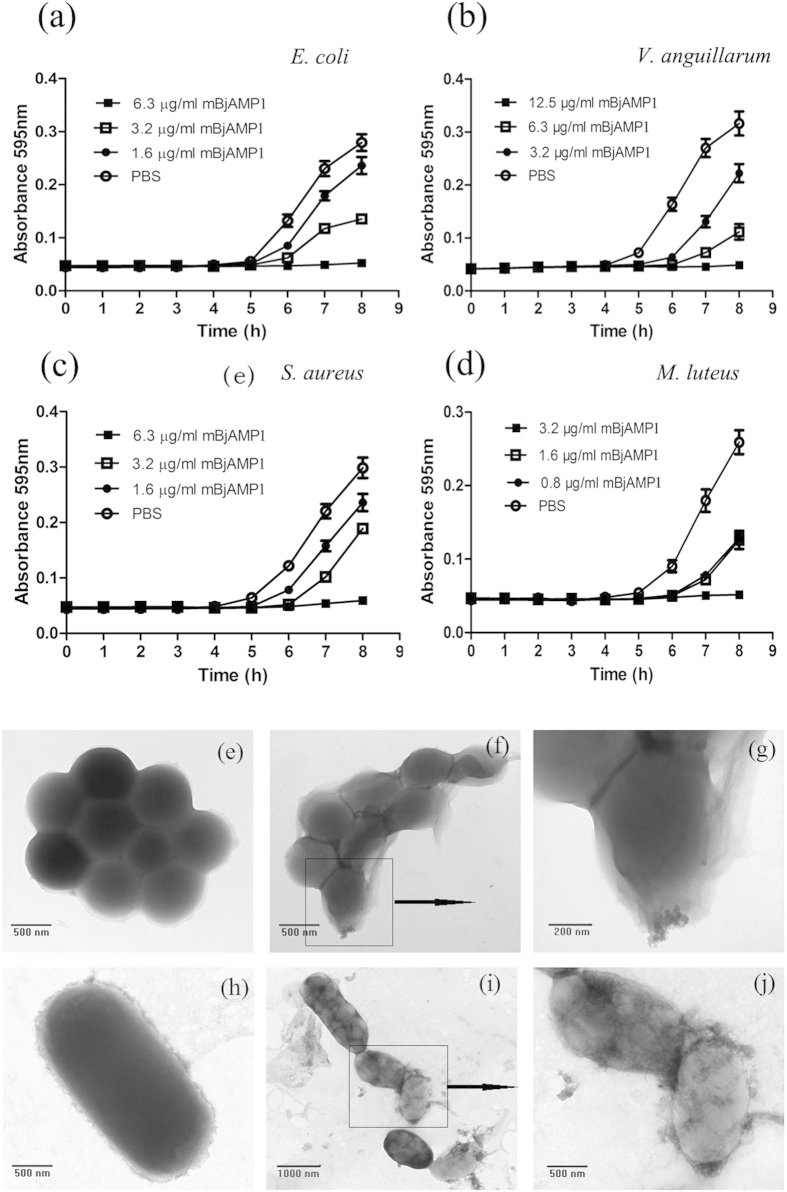
Antibacterial activity of mBjAMP1 and its effects on bacterial structure. (**a**) Antibacterial activity of mBjAMP1 against *E. coli.* (**b**) Antibacterial activity of mBjAMP1 against *V. anguillarum.* (**c**) Antibacterial activity of mBjAMP1 against *S. aureus.* (**d**) Antibacterial activity of mBjAMP1 against *M.luteus.* (**e,h**) *S. aureus* and *E. coli* incubated with PBS and observed by TEM, showing no membrane disruption/cytoplasmic thinning. (**f,i**) *S. aureus* and *E. coli* incubated with mBjAMP1 and observed, showing membrane disruption/cytoplasmic thinning. (**g,j**) High resolution of the boxes in (**f,i**).

We then tested the antimicrobial activity of mBjAMP1 against a representative set of bacterial species including 2 Gram-negative bacteria *Escherichia coli* (ATCC 25922) and *Vibrio anguillarum* (ATCC 43308) and 2 Gram-positive bacteria *Staphylococcus aureus* (ATCC 25923) and *Micrococcus luteus* (ATCC 49732). The growth of all the four bacteria was inhibited by synthesized mBjAMP1 in a dose-dependent manner (Fig. 4a–d), with minimum inhibitory concentrations (MICs) being 6.3, 12.5, 6.3 and 6.3 μg/ml for *E. coli*, *V. anguillarum*, *S. aureus* and *M. luteus*, respectively (Table 1). These indicated that mBjAMP1 is an AMP capable of inhibiting the growth of a broad spectrum of bacteria *in vitro*.

Next, we determined the effects of mBjAMP1 on fine structures of *E. coli* (a representative Gram-negative bacterium) cells and *S. aureus* (a representative Gram-positive bacterium) cells by co-incubation with mBjAMP1, followed by transmission electron microscopy (TEM) examination. It was found that mBjAMP1 caused a direct damage to the cells of *E. coli* and *S. aureus* (Fig. 4e–j), resulting in membrane disruption and cytoplasmic thinning/transparency. Cytoplasmic leakage was also been observed in both bacteria treated with mBjAMP1. These showed that mBjAMP1 is a bactericidal agent capable of directly killing the bacteria like *E. coli* and *S. aureus*.

### mBjAMP1 binds to LPS and LTA

**Figure 5:**
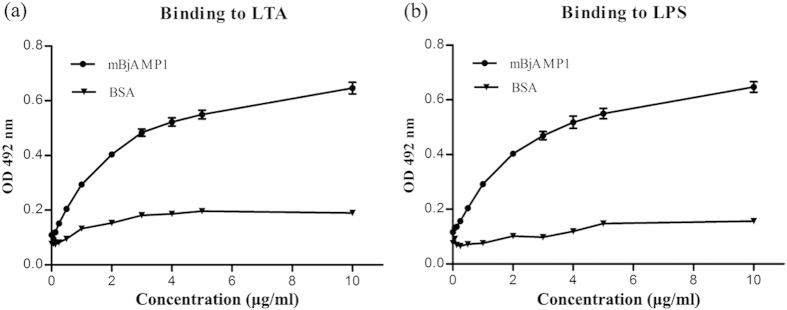
Binding of mBjAMP1 to LTA (**a**) and LPS (**b**). mBjAMP1 and BSA (control) were each applied to wells of a 96-well microplate and air-dried at 25°C overnight, and then biotin-labeled LPS or LTA was added into each well and incubated at 25°C for 3 h. The wells were each washed five times with PBST, added with 100 μl of 0.4 mg/ml Ophenylenediamine (Amresco) in the buffer consisting of 51.4 mM Na_2_HPO_4_, 24.3 mM citric acid, and 0.045% H_2_O_2_ (pH 5.0), and reacted at 37°C for 15 min. Subsequently, 50 μl of 2 M H_2_SO_4_ was added into each well to terminate the reaction, and the absorbance at 492 nm was monitored by a microplate reader. Data were expressed as mean ± SEM (n = 3). The bars represent the standard error of mean values.

Next, we tried to determine the modes of action of mBjAMP1. An enzyme linked immunosorbent assay (ELISA) was carried out to test the interaction of mBjAMP1 with LPS and LTA. As shown in Fig. 5, mBjAMP1 was able to bind to LPS and LTA, and the binding was dose-dependent and saturable, whereas bovine serum albumin (BSA) showed little affinity to LPS and LTA. These indicated that mBjAMP, in addition to being a bactericidal agent, is also a pattern recognition molecule capable of identifying LPS and LTA.

### mBjAMP1 causes membrane depolarization and permeabilization

**Figure 6:**
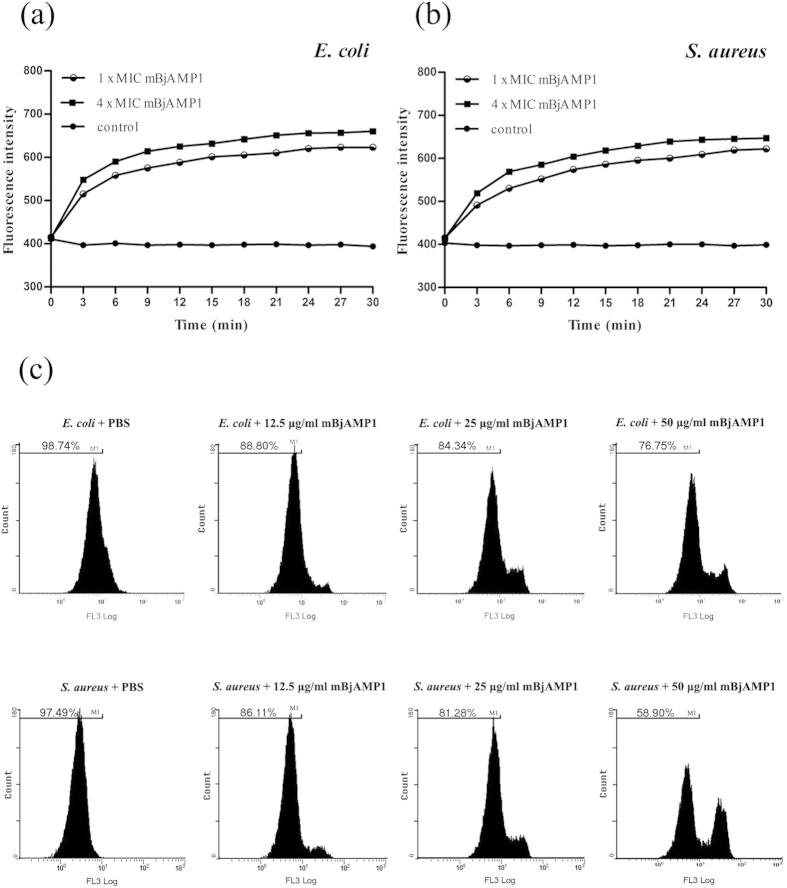
Bacterial membrane depolarization and permeabilization. (**a,b**) Depolarization of *E. coli* and *S.aureus* cell membranes were detected using DiSC_3_-5 (excitation, 622 nm; emission, 670 nm). (**c**) Effects of mBjAMP1 on the membrane integrity of *E. coli* and *S. aureus* cells analyzed by flow cytometry. The proportion of integrated cells was shown near the marker (M1).

The membrane depolarization activity of mBjAMP1 was assayed using 3,3′-dipropylthiacarbocyanine iodide (DiSC_3_-5, a potential-dependent distributional fluorescent dye). As shown in Fig. 6a,b, the fluorescence intensity of both *E. coli* and *S. aureus* cells treated with mBjAMP1 increased significantly, compared with control, indicating that mBjAMP caused depolarization of the bacterial plasma membrane. Flow cytometry was then used to test if mBjAMP1 can permeabilize the membranes of *E. coli* and *S. aureus* cells. As shown in Fig. 6c, few cells of *E. coli* and *S. aureus* in control group showed propidium iodide (PI; an intercalating agent and a fluorescent molecule) fluorescent signal, suggesting that they had intact and viable cell membranes. By contrast, a significant proportion of *E. coli* and *S. aureus* cells treated with mBjAMP1 displayed PI fluorescent signal, and the number of the bacterial cells with fluorescent signal increased with the dose of mBjAMP1, implicating that the membranes of *E*. *coli* and *S. aureus* cells were permeabilized by mBjAMP1. Collectively, these data showed that mBjAMP1 disrupted the bacterial membranes by a membranolytic mechanism including a combined action of membrane depolarization and membrane permeabilization.

### mBjAMP1 displays little cytotoxicity

**Figure 7:**
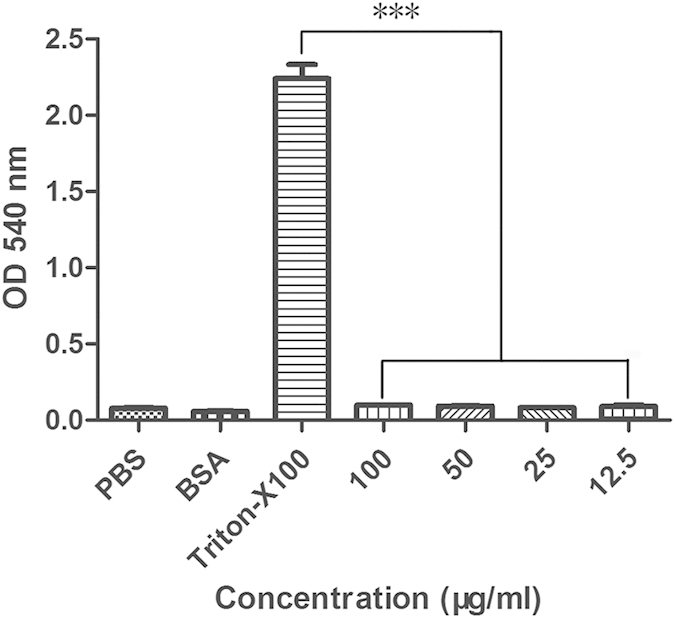
Hemolytic activity of mBjAMP1 to human blood cells (RBCs). Data were expressed as mean ± SEM (n = 3). The bars represent the standard error of the mean values. The symbol (**) indicates *p* < 0.001 compared with the Triton-X 100 treated group.

To test if mBjAMP1 was cytotoxic, its hemolytic activity on human red blood cells (RBCs) was determined. As shown in Fig. 7, mBjAMP1 showed little hemolytic activity towards human erythrocytes at all the concentrations tested. By contrast, RBCs incubated with 0.1% Triton X-100 (full lysis control) exhibited significant hemolysis. In addition, the cytotoxicity of mBjAMP1 to murine RAW264.7 cells was also tested using 3-(4,5-dimethythiazol-2-yl)-2,5-diphenyltetrazolium bromide (MTT). As shown in Table 2, mBjAMP1 was not toxic to murine RAW264.7 cells at the concentrations tested. These data showed that mBjAMP1 was non-cytotoxic to mammalian cells, suggesting that BjAMP1 has a high bacterial membrane selectivity.

## Discussion

Taking the advantage of signal sequence conservation of known AMPs to search for putative AMPs has been shown successful in the same lineages of both fish and amphibians from the EST database^15^,^17^. Here we demonstrate that such an approach is also applicable to the different lineages for we have successfully identified an amphioxus (protochordate) AMP, BjAMP1, from amphioxus databases using the signal sequence of jawless hagfish (vertebrate) HFIAP-1, a known AMP of the cathelicidin family. BjAMP1 resembles typical precursors of AMPs in that it consists of a signal peptide, anionic region and a cationic amphipathic region with a dibasic cleavage site ^75^RR^76^, common in AMPs, though it lacks the cathelin-like domain characteristic of cathelicidin family AMPs. The predicted mature AMP, mBjAMP1, comprising 21 residues with a total hydrophobic ratio of 38%, has the properties to adopt an amphipathic α-helix structure, which is commonly found in AMPs available. As mBjAMP1 shows no significant similarity to known AMPs from related sources at amino acid level, with a level of identity of <25%, thus it likely represents a new class of AMPs.

We have localized the transcripts of *Bjamp1* to distinct tissues including the gill, hepatic caecum, hind-gut, muscle and notochord. Among them, the gill, hepatic caecum and hind-gut are regarded as major immune-relevant tissues of amphioxus^23^,^24^,^25^. Importantly, challenge with LPS and LTA induces marked up-regulation of *Bjamp1* transcripts in all the tissues tested, including the hepatic caecum, notochord and muscle. These data indicated that BjAMP1 was an immune-relevant molecule associated with the immune responses of amphioxus, which was basically in line with the expectation that BjAMP1 was a potential AMP.

CD spectroscopy revealed that mBjAMP1 synthesized using standard solid-phase FMOC method showed spectra consistent with random coil structure in PBS, which underwent a coil-to-helix transition in the presence of TFE or SDS. This clearly confirms our expectation that mBjAMP1 has an α-helical properties, providing additional evidences that mBjAMP1 is an AMP. Therefore, we have synthesized mBjAMP1 and subjected to antibacterial/bactericidal activity assay, which proved that it inhibited the growth of a broad spectrum of bacteria *in vitro*, including *E. coli*, *V. anguillarum*, *S. aureus* and *M. luteus*, and causes direct damage to bacteria like *E. coli* and *S. aureus*. These data present functional evidences that showed mBjAMP1 was indeed an AMP with a broad spectrum of antibacterial/bactrericidal activity.

Potential modes of action of cationic AMPs include binding to or inserting into microbial membranes, which had fatal depolarization of the normally polarized membrane, formation of physical pores, scrambling of the usual distribution of lipids between the leaflets of the bilayer, and damage to critical intracellular targets. We showed that mBjAMP1 possessed a strong affinity for LPS from Gram-negative bacteria and LTA from Gram-positive bacteria, suggesting it can bind to the microbial surfaces via LPS and LTA. Using DiSC_3_-5, an indicator of membrane potential, we demonstrated that the membranes of microbial cells exposed to mBjAMP1 were depolarized. Moreover, when microbial cells were incubated with PI, a fluorescent dye that intercalates with DNA but cannot cross intact cell membranes, an increase in fluorescence was observed in mBjAMP1-treated cells, indicating cell death via membrane permeabilization^26^ and breach of the plasma membrane^27^. These were also corroborated by disrupted membranes and pores induced by mBjAMP1. Taken together, our results suggest that mBjAMP1 functions by a membranolytic mechanism including interaction with microbial membrane via LPS and LTA, membrane depolarization and membrane permeabilization.

A crucial point regarding the development of membranolytic antimicrobial therapeutics is that they must not destroy the membrane of mammalian cells. We showed here that compared with its antimicrobial activity, mBjAMP1 displays virtually no cytotoxic activity towards human RBCs and murine RAW264.7 cells. This implies that mBjAMP1 has a high membrane selectivity towards bacterial cells but not mammalian cells.

In summary, this study reports the identification of an uncharacterized AMP from amphioxus via *in silico* and functional analyses, presenting the first example that use of signal conserved sequence of AMPS is effective identifying potential AMPs across different animal classes. This new AMP kills a broad spectrum of microbes via membrane active mechanism, while it is non-cytotoxic to mammalian cells, rendering it a promising template for the design of novel peptide antibiotics against MDR microbes.

## Materials and Methods

### Database searching

Use of conserved signal sequences as queries has been shown to be effective in identifying putative AMPs in both teleost fish and anurans from the EST database^15^,^17^. BLASTP 2.2.31+ was used to search the non-redundant GenBank CDS translations+PDB+SwissProt+PIR+PRF for protein sequences using the sequence MKSLCVPAVLSLVLILLLDQAPTARA as probe, with search set, organism *Branchiostoma floridae* (taxid:7739) and algorithm parameters word size: 3, matrix: BLOSUM62, gap costs: existence 11, extension 1^15^,^28^. This query is the signal sequence of HFIAP-1 (GenBank AAQ04687.1), an AMP of cathelicidin family from Atlantic hagfish *Myxine glutinosa*^20^. Sequences in the output list were inspected via examining the conservation of signal peptides, and resulting 3 hits were used as queries to further search the GenBank +EMBL + DDBJ using TBLASTN 2.2.31 for translated ESTs, which resulted in identification of only one sequence (XP_002600987.1) that shows 92% identity at amino acid level to the protein encoded by BW801384.1, a cDNA sequence from Florida amphioxus *B. floridae* unpublished cDNA library. The sequence XP_002600987.1 was then used as query to search the genome database of Xiamen amphioxus *B. belcheri* (http://mosas.sysu.edu.cn/genome/index.php) for the corresponding homologous sequence, which again identified a protein sequence (ID: 003420R.t2) showing 84% and 92.8% identity to the hypothetical protein XP_002600987.1 and translated BW801384.1, respectively. These analyses suggested that the cDNA sequence BW801384.1 from *B. floridae* corresponded to AAQ04687.1 from Atlantic hagfish, and on translation showed the characteristics of a putative AMP.

### Cloning of *Bjamp1* cDNA

Based on analyses above, we tried to clone the homologue of BW801384.1, hereafter named as *Bjamp1*, from Qingdao amphioxus *B. japonicum*. Total RNAs were extracted from *B. japonicum* with Trizol (Invitrogen) according to the manufacturer’s instructions. After digestion with recombinant RNase-free DNase (TaKaRa) to eliminate the genomic contamination, the first-strand cDNA was synthesized with reverse transcription system (Promega) using oligo d(T) primer, and used as PCR template. The fragment of *Bjamp1* was amplified by PCR with the primer pairs P1-F and P1-R (Table 3) that were designed using Primer Premier 5.0 program on the basis of relative sequences identified in *B. floridae* genome database (http://genome.jgi-psf.org/Brafl1/Brafl1.home.html) and in *B. belcheri* genome database (http://mosas.sysu.edu.cn/genome/index.php). After determination of the partial cDNA sequence, rapid amplification of cDNA ends (RACE) was employed to obtain the full-length cDNA. The gene-specific primer pairs P2-F and P3-F (Table 3) were used in RACE reactions for the cloning of 3’-end cDNAs. The 3’-RACE-Ready cDNAs were synthesized from the total RNAs using the 3’ Full RACE Core Set (TaKaRa, Dalian, China) according to the manufacturer’s instructions. The products of 3’-RACE were gel-purified, sub-cloned and sequenced, and *Bjamp1* cDNA was obtained by assembling the overlapping sequences.

### Sequence analyses

The cDNA obtained was analyzed for coding probability with the EditSeq in DNASTAR software package (DNASTAR Inc., Madison, WI, USA). Sequence comparison was performed using the MegAlign program by CLUSTAL W method^29^ in DNASTAR software package. The SMART program (http://smart.embl-heidelberg.de/) was used to predict the functional sites and domains and the SignalP 4.0 (http://www.cbs.dtu.dk/services/SignalP/) used to predict the signal peptide. The molecular mass (MW) and isoelectric point (pI) of the mature peptide were calculated using ProtParam (http://www.expasy.ch/tools/protparam.html). PeptideCutter (http://web.expasy.org/peptide_cutter/) and CAMP (http://www.camp.bicnirrh.res.in/predict/) were used to predict the cut site and the position of a predicted mature AMP. The three-dimensional (3D) structure was generated by QUARK ONLINE (http://zhanglab.ccmb.med.umich.edu/QUARK/). The Jpred program (http://www.compbio.dundee.ac.uk/www-jpred/index.html) was used to predict the secondary structure. Antimicrobial Peptide Calculator and Predictor at APD (http://aps.unmc.edu/AP/main.php) was used to predict the total hydrophobic ratio.

Helical wheel of the predicted mature AMP was generated using the internet site “helical wheel projection” (http://rzlab.ucr.edu/scripts/wheel/wheel.cgi) created by Armstrong and Zidovetzki^30^. By default the output presents the hydrophilic residues as circles, hydrophobic residues as diamonds, potentially negatively charged residues as triangles, and potentially positively charged residues as pentagons. Hydrophobicity was color coded as well: the most hydrophobic residue was green, and the amount of green was decreasing proportionally to the hydrophobicity, with zero hydrophobicity coded as yellow. Hydrophilic residues were coded red with pure red being the most hydrophilic (uncharged) residue, and the amount of red decreasing proportionally to the hydrophilicity. The potentially charged residues were light blue.

### Quantitative real-time PCR

qRT-PCR was used to examine the expression patterns of *Bjamp1* in the different tissues of *B. japonicum*. Total RNAs were extracted with Trizol (Invitrogen) from the different tissues including the gill, hepatic caecum, hind-gut, testis, ovary, notochord and muscle. After digestion with RNase-free DNase (Promega) to eliminate the genomic contamination, cDNAs were synthesized and used as template for reverse transcription system using oligo d(T) primer. The PCR primers P4-F and P4-R specific of *Bjamp1* and the primers P5-F and P5-R specific of EF1α gene (Table 3) were designed using the primer premier 5.0 program. The reaction mixture (final volume 20 μl) consisted of 10 μl of SYBR Premix ExTaq™ (Tli RNaseH Plus), 0.4 μl of ROX Reference Dye II, 0.5 μl of template and 200 nM of each sense and antisense primers. The EF1α gene was chosen as the reference for internal standardization. qRT-PCR experiments were conducted in triplicate. The amplification was performed on ABI 7500 real-time PCR system (Applied Biosystems) at 95 °C for 15 s, followed by 40 cycles of 95 °C for 5 s, 60 °C for 15 s, and 72 °C for 30 s. Melting curve analysis of amplification products was performed at the end of each PCR to confirm that only one PCR product was amplified and detected. The expression levels of *Bjamp1* relative to that of EF1α gene were calculated by the comparative CT method (2^−ΔΔCT^)^31^.

qRT-PCR was also performed to assay the expression profiles of *Bjamp1* in response to challenge with the microbial signature molecules LPS and LTA, respectively, as described previously^32^. Briefly, adult *B. japonicum* were exposed to sterilized seawater with 10 μg/ml of LPS (Sigma, USA) or LTA (Sigma, USA), and sampled at 0, 2, 4, 8, 12, 24, 36 and 48 h post exposure. The different tissues including the hepatic caecum, muscle and notochord were dissected out of the animals, and total RNA extraction, cDNA synthesis and qRT-PCR were performed as above.

### Peptide synthesis

As analyses by PeptideCutter and CAMP predicted a mature AMP, mBjAMP1, consisting of 21 amino acids (NLCASLRARHTIPQCRKFGRR), it was thus synthesized by Shanghai Sangon Biological Engineering Technology & Services Co., Ltd, using standard solid-phase FMOC method, and used for functional characterization. Because C-terminal amidation is common in AMPs^33^, the C-terminus of the synthetic mature peptide, designated mBjAMP1, was therefore amidated (NLCASLRARHTIPQCRKFGRR-NH_2_). The peptide synthesized was purified to >95% by high-performance liquid chromatography (HPLC) and verified using a mass spectrometer (lcms-2010a, Shimadzu, Japan) as well as by MS/MS to further confirm the sequence. The peptide was then dissolved in 1% (v/v) 2,2,2-trifluoroethanol water solution (2 mg/ml) and stored at −80 °C till used.

### Circular dichroism spectroscopy

The propensity of mBjAMP1 to assume a helical conformation was probed by CD spectroscopy Jasco J-810 spectropolarimeter (Tokyo, Japan), in a 1-mm path-length quartz cells and with a peptide concentration of 100 μg/ml^15^,^34^. Spectra were measured in the presence of PBS, increasing amounts TFE and SDS micelles at room temperature. The spectra were averaged over three scans.

### Antimicrobial activity assay

The assay for antimicrobial activity of mBjAMP1 against the Gram-negative bacteria *Escherichia coli* (ATCC 25922) and *Vibrio anguillarum* (ATCC 43308) and the Gram-positive bacteria *Staphylococcus aureus* (ATCC 25923) and *Micrococcus luteus* (ATCC 49732) was performed as described previously^35^ with slight modification. In brief, all the four strains of the bacteria were cultured in LB medium to mid-logarithmic phase, and then collected by centrifugation at 6000 g at room temperature for 10 min. The bacterial pellets were washed in PBS three times, re-suspended in PBS and adjusted to a density of 10^5^ cells/ml. mBjAMP1 solution was diluted two-folds serially (starting at 200 μg/ml) with PBS, giving concentrations of 200, 100, 50, 25, 12.5, 6.3, 3.2 μg/ml, respectively. Aliquots of 50 μl mBjAMP1 solution were each mixed with 50 μl of the bacterial suspension, with final concentrations of mBjAMP1 being 100, 50, 25, 12.5, 6.3, 3.2 and 1.6 μg/ml. For control, 50 μl PBS was mixed with 50 μl of the bacterial suspension. All the mixtures were pre-incubated at 25 °C for 2 h, and then were each allocated into 3 wells (30 μl/well) in a sterile 96-well plate, into which 170 μl fresh LB medium per well was added. After incubation at 37 °C for 10 h, the absorbance was measured at 595 nm once every hour using a Microplate Reader (Multiskan GO; Thermo Scientific). The MIC was defined as the lowest peptide concentration at which the growth of the respective bacterium was completely inhibited.

### Transmission electron microscopy

TEM was performed to test the effect of mBjAMP1 on bacteria cell walls by the method of Hu *et al.*^36^. In brief, aliquots of 500 μl of *E. coli* (a representative of Gram-negative bacteria) and *S. aureus* (a representative of Garm-positive bacteria) suspensions containing 5 × 10^7^ cells/ml were mixed with 500 μl of mBjAMP1, giving a final concentration of 50 μg/ml (8 × MIC, see Table 1 below), or with 500 μl of PBS alone as control. The mixtures were incubated at 25 °C for 2 h, fixed in 2.5% glutaraldehyde in 100 mM PBS (pH 7.4), and then dropped onto 400-mesh carbon-coated grids and allowed to stand at room temperature for 3 min for negative staining. Excess fluid was removed by touching the edge of filter paper. The grids were then put into 2% phosphotungstic acid for 3 min and dried by filter paper. Observation was performed under a JEOL JSM-840 transmission electron microscope.

### Assay for binding of mBjAMP1 to LPS and LTA

An ELISA was performed to test if mBjAMP1 could bind to LPS and LTA. Both LPS and LTA were labeled with biotin hydrazide (Sigma-Aldrich) as previously described^37^. Aliquots of 50 μl of 50 μg/ml mBjAMP1 or BSA (control) were applied to each well of a 96-well microplate and air-dried at 25 °C overnight. The plate was incubated at 60 °C for 30 min to fix the peptide, and then each well was blocked with 100 μl of 1 mg/ml BSA in PBS (pH 7.4) at 37 °C for 2 h. After washing five times with 200 μl of PBS (pH7.4) containing 0.1% Tween-20 (PBST), a total of 50 μl PBS containing 0.1 mg/ml BSA and different concentrations of biotin-labeled LPS (0, 0.063, 0.125,0.25, 0.5, 1, 2, 3, 4, 5 and 10 μg/ml) or LTA (0, 0.063, 0.125,0.25, 0.5, 1, 2, 3, 4, 5 and 10 μg/ml) was added into each well and incubated at 25 °C for 3 h. The wells were each washed five times with 200 μl of PBST, added with 100 μl of streptavidin-HRP (CWBIO) diluted to 1: 3000 with 0.1 mg/ml BSA in PBS and incubated at 25 °C for 1 h. Subsequently, the wells were each washed five times with 200 μl of PBST, added with 100 μl of 0.4 mg/ml Ophenylenediamine (Amresco) in the buffer consisting of 51.4 mM Na_2_HPO_4_, 24.3 mM citric acid, and 0.045% H_2_O_2_ (pH 5.0), and reacted at 37 °C for 15 min. Finally, 50 μl of 2 M H_2_SO_4_ was added into each well to terminate the reaction, and the absorbance at 492 nm was monitored by a microplate reader (Multiskan GO; Thermo Scientific).

### Membrane depolarization assay

The assay for membrane depolarization activity of mBjAMP1 was performed with the membrane potential-sensitive dye DiSC_3_-5 (DiSC_3_-5; Sigma-Aldrich) and the Gram-negative bacterium *E. coli* and the Gram-positive bacterium *S. aureus*^34^. The bacterial cells in the mid-logarithmic phase were harvested by centrifugation at 6000 g for 10 min), washed in 5 mM HEPES buffer (pH7.3) containing 20 mM glucose, and re-suspended in 5 mM HEPES buffer (pH7.3) containing 20 mM glucose and 100 mM KCl to an OD_600_ of 0.05. A stock solution of diSC_3_-5 was added to the bacterial suspensions, yielding a final concentration of 0.5 μM, and incubated at room temperature for 30 min to get a steady baseline of fluorescence intensity. The bacterial suspensions were then mixed with mBjAMP1 solution, giving the desired concentrations of 1 × MIC and 4 × MIC of mBjAMP1 (see Table 1 below). HEPES buffer (pH7.3) containing 20 mM glucose was used as control. Changes in fluorescence intensity were continuously recorded for 30 min with a TECAN-GENios plus spectrofluorimeter at an excitation wavelength of 622 nm and an emission wavelength of 670 nm.

### Assay for permeability of microbial cells

The assay for permeabilization activity of mBjAMP1 was carried out using *E. coli* and *S. aureus* cells by flow cytometry as described by Hu *et al.*^36^. The bacteria were cultured in LB medium to mid-logarithmic phase, and harvested by centrifugation at 6000 g for 10 min. After washing three times with PBS, the bacterial pellets were suspended in PBS, adjusted to a density of 1 × 10^6^ cells/ml, and mixed with mBjAMP1 solution, giving final concentrations of 12.5 μg/ml (2 × MIC; see Table 1) and 25 μg/ml (4 × MIC; see Table 1), respectively. For control, the bacteria were mixed with PBS alone. The mixtures were incubated at 37 °C for 1 h, and fixed with 10 μM of PI solution under dark at 4 °C for 15 min. The bacterial cells staining by PI was examined using a FC500 MPL flow cytometer (Beckman). Data was analyzed using WinMDI v.2.9 software (Scripps Research Institute, San Diego, CA).

### Hemolytic activity assay

Human red blood cells (RBCs) were used to test the hemolytic activity of mBjAMP1 as described Hu *et al.*^36^. Healthy human blood was obtained into a EDTA anticoagulant tube, and RBCs were collected by centrifugation at room temperature at 1000 g for 10 min. After washing three times with PBS, RBCs were suspended in PBS to give a concentration of 4% (v/v). An aliquot of 200 μl RBCs suspension was mixed with 200 μl of mBjAMP1 solution, giving final concentrations of 12.5, 25, 50 and 100 μg/ml. After incubation at 37 °C for 1 h, the mixtures were centrifuged at room temperature at 1000 g for 10 min. The supernatants were collected and added into a 96-well plate. The absorbance was measured at 540 nm under a microplate reader (Multiskan GO; Thermo Scientific). RBCs incubated with PBS alone, BSA solution (100 μg/ml), 0.1% Triton X-100 solution served as blank, negative and positive controls. Each treatment was performed in triplicate.

### MTT assay

To test if mBjAMP1 is cytotoxic to murine RAW264.7 cells, MTT assay was performed as described by Hu *et al.*^36^. RAW264.7 cells were suspended in serum-free DMEM and aliquots of 180 μl of the cell suspension (1 × 10^6^ cells/ml) were sampled into a 96-well plate and cultured at 37 °C with 5% CO_2_ for 2 h. Subsequently, aliquots of 200 μl of mBjAMP1 in serum-free DMEM were added to each well, giving final concentrations of 12.5, 25, 50 and 100 μg/ml, respectively, incubated for another 4 h, and then 20 μl of MTT solution (5 mg/ml in PBS) was added into each well. After incubation for 4 h, the medium was removed and 150 μl of dimethyl sulfoxide (DMSO) was added. The absorbance at 492 nm was measured under a microplate reader. The cells treated with DMEM alone served as control. The percent viability against the control was calculated as follows: (OD of treated groups/OD of control groups) ×100% (n = 3).

### Statistical analysis

All the experiments were performed in triplicate, and repeated three times except TEM experiment. Statistical analyses were performed using the GraphPad Prism 5. The significance of difference was determined by two-way ANOVA. Difference at p < 0.05 was considered significant. All the data were expressed as mean ± SEM.

## Additional Information

**How to cite this article**: Liu, H. *et al.* Identification of a novel antimicrobial peptide from amphioxus *Branchiostoma japonicum* by *in silico* and functional analyses. *Sci. Rep.* **5**, 18355; doi: 10.1038/srep18355 (2015).

## Supplementary Material

Supplementary Information

## Figures and Tables

**Table 1:** Minimum inhibitory concentration (MIC) of mBjAMP1 against bacteria.

Bacterium	*E. coli.*	*V. anguillarum.*	*S. aureus*	*M. luteus*
MIC (μg/ml)	6.3	12.5	6.3	6.3

**Table 2:** The percent viability of RAW264.7 cells in the presence of mBjAMP1.

mBjAMP1 concentration (μg/ml)	Percent viability (%)
0	100
12.5	100 ± 5
25	117 ± 2
50	99 ± 7
100	100 ± 7

**Table 3:** Sequences of the primers used in this study.

Primer	Primer sequence (5′–3′)
P1-F	ATGGCCCGCCTTGCAGTGTTCGT
P1-R	CTAGCGGCGGCCAAACTTTTTGCA
P2-F	ATGGCCCGCCTTGCAGTGTTCGT
P3-F	TGTGCGTCGTCACTTTGCTAC
P4-F	AGGGCAGTGGACAAGGCAGAA
P4-R	TCGTCCTCGCTGGCTGGTAGC
P5-F	TGCTGATTGTGGCTGCTGGTACTG
P5-R	GGTGTAGGCCAGCAGGGCGTG
