# Supplementary material for: Identification of a novel antimicrobial peptide from amphioxus Branchiostoma japonicum by in silico and functional analyses
Source: Sci Rep. 2015 Dec 18;5:18355. doi: 10.1038/srep18355 (PMC4683396; doi:10.1038/srep18355)
Supplement: Supplementary Information [file srep18355-s1.doc]

**Identification of a novel antimicrobial peptide from amphioxus *Branchiostoma japonicum* by *in silico* and functional analyses**

Haohan Liu, Miaomiao Lei, Xiaoyuan Du, Pengfei Cui and Shicui Zhang*

*Laboratory for Evolution & Development, Institute of Evolution & Marine Biodiversity and Department of Marine Biology, Ocean University of China, Qingdao 266003, China*

*Correspondence author

Dr. Shicui Zhang

Room 205, Ke Xue Guan, 5 Yushan Road, Ocean University of China,

Qingdao 266003, China

Tel.: +86 532 82032787

E-mail: [sczhang@ouc.edu.cn](mailto:sczhang@ouc.edu.cn)

**Supplementary Figure 1.** The open reading frame (ORF) and deduced amino acid sequences of *B. floridae* antimicrobial peptide 1 (*Bfamp1*) and *Bjamp1.* (a) *Bfamp1* (accession number in GenBank: BW801384.1). (b) *Bjamp* (accession number in GenBank: KR779875). The predicted signal peptide is underlined in red, and the predicted mature peptides is underlined in blue.

**
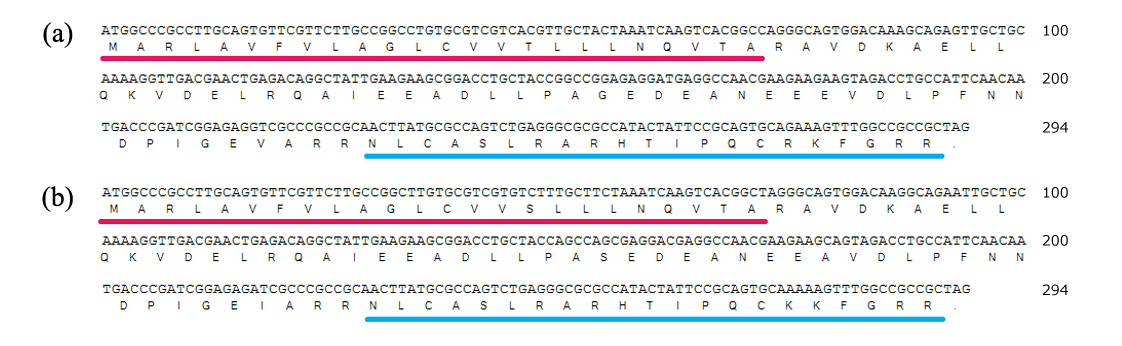
**

**Supplementary Figure 2.** Alignment of BjAMP1 with its homologues from *B. floridae* and *B. belcheri.* The symbol (*) shows the different amino acid residues in the predicted mature peptide.

**
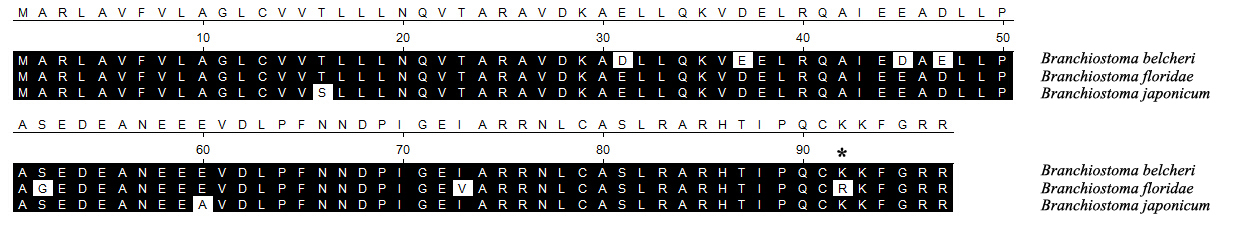
**

**Supplementary Figure 3**. Confirmation of the sequence of mBjAMP1 synthesized.

**Supplementary Figure 3a**. MS Spectrum

**
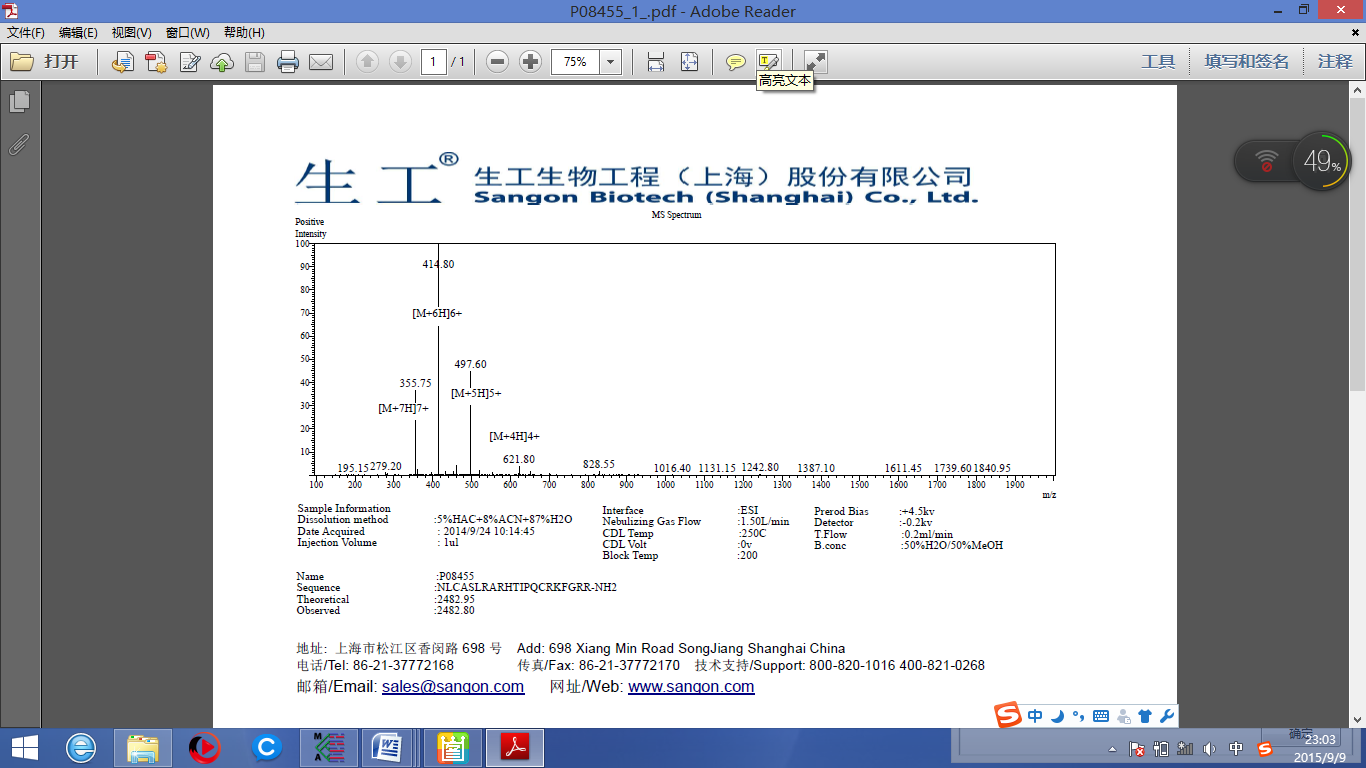
**

**Supplementary Figure 3b**. MS/MS Spectrum

**
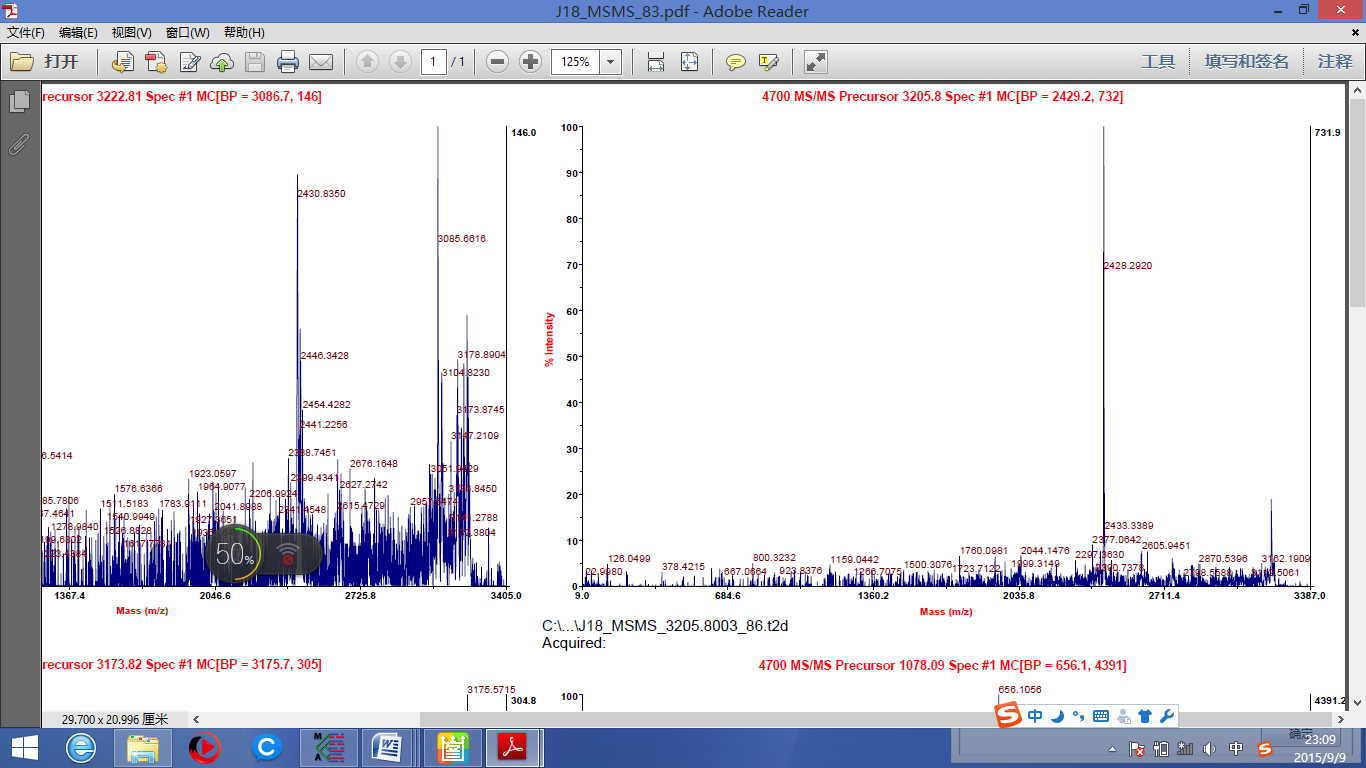
**

**Supplementary Table 1.** Five peptides most similar to mBjAMP1 in APD.

| **Number**  **in APD** | **Similarity** | **Identity** | **Length** | **Name/Class** | **Source** | **Sequence** |
| --- | --- | --- | --- | --- | --- | --- |
| AP02278 | 38.46% | 14.3% | 24 | Brevinin-1SN1 | Frog | FLPAVLKVAAHILPTAICAISRRC |
| AP01908 | 37.5% | 20.0% | 20 | Ranacyclin-B-RL1 | Frog | AALRGCWTKSIPPKPCPGKR |
| AP00491 | 37.5% | 20.0% | 20 | Ranacyclin B5 | Frog | AALRGCWTKSIPPKPCSGKR |
| AP01291 | 37.5% | 25.0% | 20 | Odorranain-B1 | Frog | AALKGCWTKSIPPKPCFGKR |
| AP00035 | 37.03% | 14.3% | 26 | Plantaricin A | *Lactobacillus plantarum* | KSSAYSLQMGATAIKQVKKLFKKWGW |
